# Efficacy and Safety of Shatavari Root Extract for the Management of Menopausal Symptoms: A Double-Blind, Multicenter, Randomized Controlled Trial

**DOI:** 10.7759/cureus.57879

**Published:** 2024-04-08

**Authors:** Vani S Gudise, Meenakshi P Dasari, Siva Sai K Kuricheti

**Affiliations:** 1 Obstetrics and Gynecology, Good Life Hospital, Vijayawada, IND; 2 Obstetrics and Gynecology, Ravi Nursing Home, Guntur, IND; 3 Auyrvedic Medicine, Rasashastra and Bhaishajya Kalpana, Sree Jeevana Ayurveda, Vijayawada, IND

**Keywords:** regensburg insomnia scale, utian quality of life, menopausal symptoms, shatavari, ayurveda, asparagus racemosus, menopause

## Abstract

Background

Menopause is a physiological state that occurs in all women and refers to the halt of the reproductive phase. The cessation of the reproductive phase occurs through various stages and presents different symptoms such as hot flashes, night sweats, insomnia, anxiety, depression, and irritability. Such pre- and post-menopausal symptoms may affect the daily activities and production capacities of women, impacting the quality of life (QoL) of women. Hormone replacement therapy (HRT) is primarily used to manage menopausal symptoms. However, various side effects have been reported related to HRT. Therefore, women are choosing alternative medicine such as Ayurveda that can benefit them with less or no adverse effects. Shatavari (*Asparagus racemosus*) is known in Ayurveda as an effective medicinal plant source for various women’s health remedies since ancient times. This study aimed to evaluate the safety and efficacy of the Ayurvedic Shatavari formulation on menopausal symptoms compared to the placebo.

Methodology

This is a randomized, double-blinded, multicenter, placebo-controlled, clinical study. Altogether, 70 patients were randomized to two groups, i.e., the test group (active group) and the placebo group (microcrystalline cellulose), with 35 participants in each group.

Results

The study outcomes showed a positive and significant effect of the active test ingredient over the placebo in terms of reduction in hot flashes, night sweats, insomnia, anxiety, nervousness, vaginal dryness, and loss of libido. The Utian QoL improved significantly in the test group compared to the placebo group. No significant adverse events were recorded in the test group, suggesting the safety of this formulation.

Conclusions

The test compound could be a safe alternative to modern drugs. The findings of this study support the traditional use of Shatavari. Further clinical and pharmacological studies with longer duration and larger and more diverse sample sizes are required to understand the generalized effect of Shatavari root extract in menopausal women.

## Introduction

Menopause is a pivotal and inevitable physiological milestone in the lives of women [1]. The global population of postmenopausal women is on the rise, constituting 26% of all females under the age of 50 [2]. Notably, there is a 21% increase in the occurrence of early-age menopause. The sudden or gradual decline in estrogen levels creates an imbalance in the hypothalamic-pituitary-ovarian (HPO) axis. This can lead to failure of endometrial development causing irregular menstrual cycles and progressively contributing to the halt of periods [3].

During menopause, the intricate interplay of changing hormonal levels, specifically estrogen and progesterone, renders women more vulnerable to a spectrum of symptoms. These symptoms encompass hot flashes, vaginal dryness, disturbed sleep, insomnia, urogenital infections, osteoporosis, early onset of coronary heart disease, anxiety, depression, diabetes, and cognitive difficulties. Collectively, these symptoms are comprehensively categorized as menopausal symptoms [4]. Approximately, 75% of women experience perimenopausal or menopausal symptoms due to estrogen deficiency. The transition from a perimenopausal state to menopause is often troublesome, mainly because of menopausal symptoms. By 2025, there will be approximately 1.1 billion menopausal women globally [5].

Menopausal symptoms may be mitigated to a certain degree through the use of local or systemic exogenous estrogen administration via hormone replacement therapy (HRT) [6]. However, the utilization of HRT is linked with increased risks of complications, such as breast cancer, heart disease, and thromboembolism, as well as other potential side effects [7]. Hence, an alternative therapy devoid of such risks or reduced risks is warranted to manage menopausal symptoms. In a systematic review, it was reported that across all surveys, more than 50% of the women used complementary and alternative medicine to treat their menopausal symptoms specifically [8]. Ayurvedic treatment principles have huge potential for managing menopausal symptoms. Menopause can be studied under the concepts of Jaravaydhi, Rajonivrutti, Dhatukshayja, and Vatavruddhi in Ayurvedic classics [9]. The main principles of treatment are Rasayana Chikitsa and Pitta Vatashamana, which can be attained with certain herbal preparations. Such Ayurvedic formulations, when used wisely, can help relieve menopausal symptoms and related aging issues [10].

Shatavari (*Asparagus racemosus* *Willd*.), a herb, has been widely used in Ayurveda but without much empirical evidence for its effectiveness [11]. Shatavari predominantly balances Pitta dosha followed by Vata dosha. Pitta dosha is the biological energy responsible for digestion and balancing or regulating all metabolic and hormonal activities in the human body [12]. Shatavari contains phytoestrogens, compounds known for their estrogen-like effects, which play a crucial role in alleviating the discomfort associated with menopause. Steroidal saponins, Shatavarins, are the principal bioactive constituents of Shatavari root. Others include alkaloids, quercetin, and glycosides of quercetin [13]. The traditional use of Shatavari suggests potential benefits in managing women’s hormonal disorders, making it a noteworthy candidate for further investigation and research.

The test product (Aspurūs) is a standardized herbal root extract of Shatavari. This clinical study was conducted to assess the safety and efficacy of the extract in the management of menopausal symptoms in humans.

## Materials and methods

Study design

**Figure 1:**
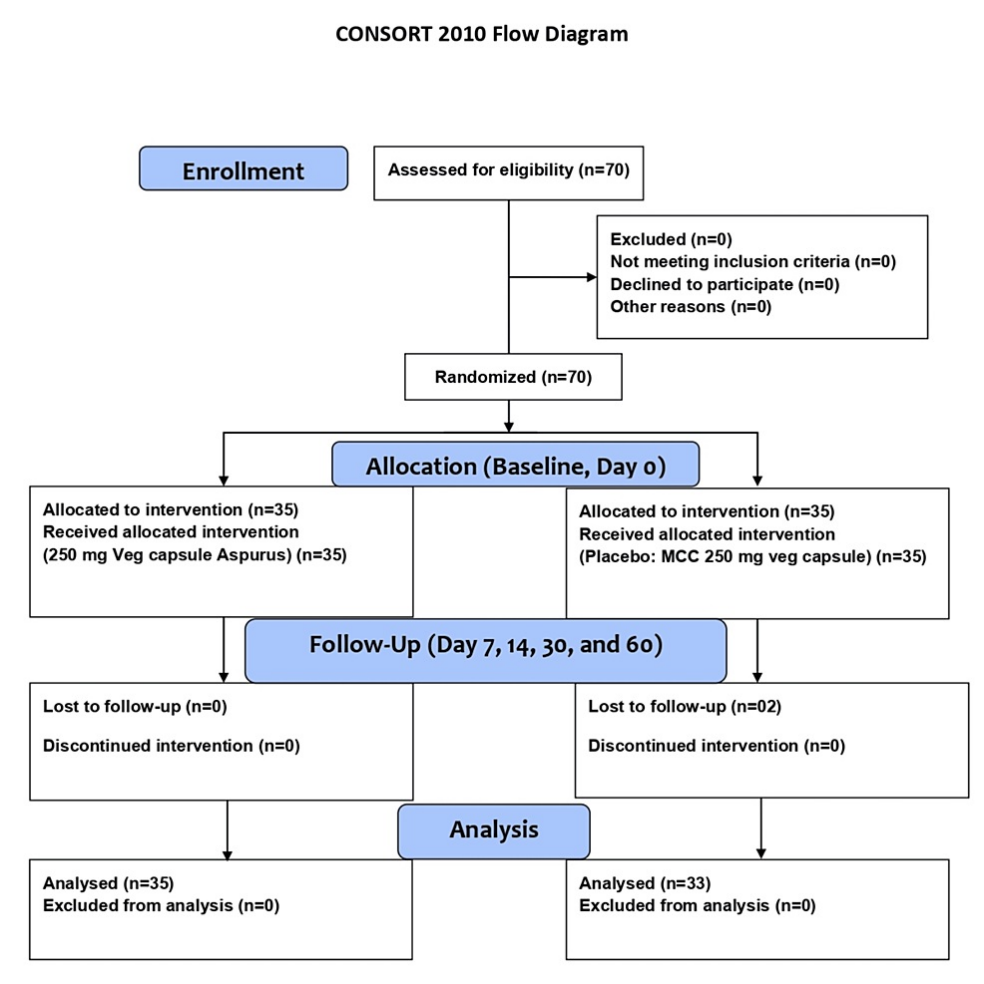
The CONSORT flowchart of the study design.

This was an eight-week, multicenter, interventional, prospective, randomized, double-blind, placebo-controlled, parallel clinical trial structured to compare the safety and efficacy of the test active ingredient with the placebo in the management of menopausal symptoms and regulation of the HPO axis in pre and postmenopausal women (Figure 1). The trials were conducted at two locations in Vijayawada and Guntur, Andhra Pradesh, India. The study was conducted in Good Life Hospital in Vijayawada (n = 40) and Ravi Nursing Home in Guntur (n = 30), Andhra Pradesh, from August 10, 2023, to October 26, 2023.

Ethical considerations

The study was conducted following the clinical trial ethical requirements of the Declarations of Helsinki. Approval from the Independent Ethics Committee was obtained from Fusion Clinical Research (vide letter no: ECR/375/Indt/AP/2023, dated: 08.04.2023), Vijayawada.

Test product description

The test product used for the present study was Aspurūs™. This is a full-spectrum Shatavari that focuses on delivering a standardized *Asparagus racemosus* root extract. The extraction was done using a unique proprietary method, and the root extract was standardized to the phytoconstituents of total Shatavarins (steroidal saponins) of 5% with high-performance thin-layer chromatography. The allergen-free test product was tested for heavy metals such as lead, arsenic, cadmium, and mercury following the Association of Official Agricultural Chemists 2015.01 standard methodology.

Study protocol and participant selection criteria

The inclusion criteria were pre and postmenopausal women aged between 40 and 65 years who were experiencing menopausal symptoms such as hot flashes, night sweats, anxiety, fatigue, depression, insomnia, and mood swings, as diagnosed by the study investigator.

The exclusion criteria were pregnant women or lactating women, women who were actively trying to conceive, participants who had undergone hysterectomy, and women who were on HRT or any other herbal treatments for the management of menopausal symptoms. Participants with a history of any serious illnesses in the last three months or any history of current alcohol or substance abuse were excluded. Other exclusion criteria included women with an allergy or sensitivity to any ingredients in the study treatment, those who had participated in any other clinical studies in the past three months, or those who intended to participate in other clinical studies parallelly.

A total of 70 women who met all the inclusion and exclusion criteria and provided informed consent were enrolled in the trial. They were randomly assigned to either the active (test product group) or placebo (microcrystalline cellulose (MCC)) group using a computer-generated schedule. All recruited women were administered one capsule of 250 mg containing either the active (test product) (Shatavari root extract) or placebo (MCC containing all excipients except the active ingredient) twice a day for 60 days (eight weeks). Detailed demographic, medical, and medication histories were obtained from the participants. Vital parameters were collected on day 1, day 30, and day 60 and analyzed. Laboratory tests to determine the levels of serum estradiol and progesterone were performed.

Outcome measures

Primary Outcome Measures

The changes in the total Utian quality of life (UQoL) scores on day 30 and day 60 were compared to the baseline scores.

Secondary Outcome Measures

Evaluation of the changes in specific parameters such as individual domain scores of UQoL, i.e., occupational UQoL, health UQoL, emotional UQoL, and sexual UQoL; the score of all three (depression, anxiety, and stress) domains of Depression, Anxiety and Stress Scale - 21 Items (DASS-21); the total score of Regensburg Insomnia Scale (RIS) along with RIS sub-scales (poor sleep depth, poor sleep quantity, fearful focus on insomnia, hypnotics and poor daytime functioning; question 1 (How many minutes do you need to fall asleep?), and question 2 (How many hours do you sleep during the night?)); menopausal symptoms using a five-point Likert scale; and serum estradiol and progesterone. The Treatment Satisfaction Questionnaire (MS-TSQ) was assessed at the day 30 visit and the final visit (day 60). Changes in any vital parameters were also examined.

Safety Outcomes

The safety of the trial intervention was evaluated by recording the incidence of adverse events on every scheduled follow-up visit. All adverse events during the study timeline were recorded and monitored following the Good Clinical Practice guidelines.

Statistical analysis

After a complete follow-up of participants, data were pooled and analyzed statistically. All results were analyzed using rio, gtools, ggplot2, dplyr, and stats packages in R software (version 4.3.2). For continuous variables with normally distributed data within groups, a paired t-test was used, and the results are presented as mean differences with respective p-values.

For non-normally distributed data within the group, the Wilcoxon signed-rank test was used, and the findings are presented as median with respective p-values. The intragroup comparison was done by repeated-measures analysis of variance. The intergroup comparison was performed by an unpaired t-test for normally distributed data and the Mann-Whitney U test for non-normally distributed data. The level of statistical significance was considered at p-values <0.05. For categorical variables, the frequency and percentage of the population are presented. A descriptive comparison was done to differentiate the intra and intergroup treatment effects.

## Results

Primary outcome

**Figure 2:**
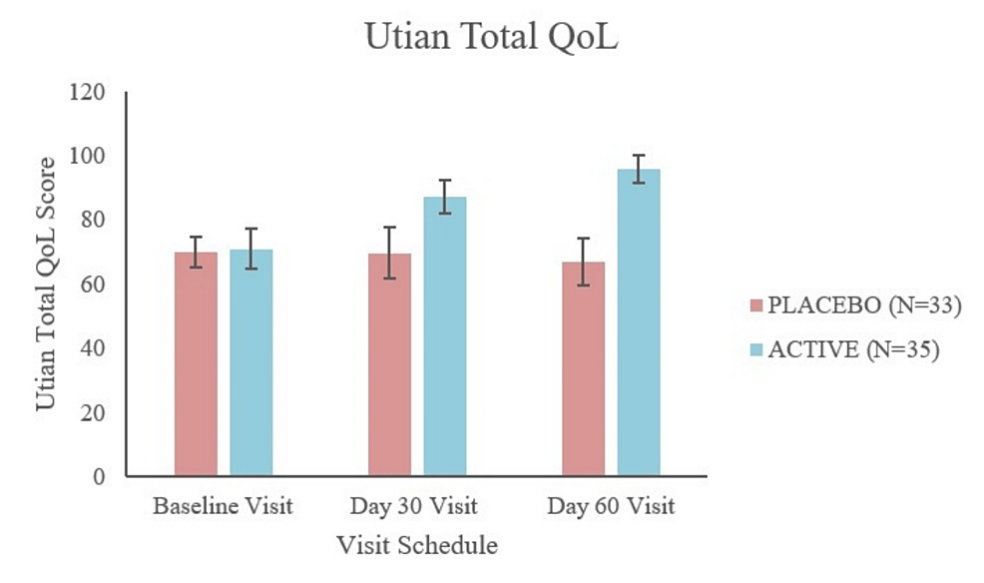
Utian total quality of life (QoL) scores. The baseline scores and the respective changes on day 30 and day 60 for the placebo and active test groups are presented.

The primary outcome of the study was to observe the change in the total UQoL score compared to the baseline. Figure 2 presents a summary of the UQoL scale as measured on day 30 and day 60. An increase in the total score by 16.46 (23.19%) and 24.94 (35.13%) on day 30 and day 60, respectively, was observed in the active (test product) group. Statistically significant intragroup differences were observed on both day 30 (p < 0.0001) and day 60 (p < 0.0001).

Secondary outcome

The active (test group) group was found to be better in therapeutic response compared to the placebo (MCC) group in each of the occupational UQoL score, health UQoL score, emotional UQoL score, and sexual UQoL score (Table 1). There was a significant change in the occupational UQoL score, health UQoL score, emotional UQoL score, and sexual UQoL score with a comparative p-value of 0.2063, 0.5678, 0.2751, and 0.7993, respectively, at baseline visit to <0.0001 at the final visit (visit 5) in the active group. An intragroup significant change (p < 0.0001) in the mean scores of all the domains of UQoL in the active group was observed.

**Table 1:** Summary statistics of Utian quality of life (UQoL) scale: health, emotional, sexual, and occupational quality of life.

Summary statistics of the Utian quality of life (UQoL) scale				
Parameter	Visit	Placebo (N = 33)	Active (Aspurūs) (N = 35)	Intergroup P-Value
Occupational QoL	Baseline visit	20.91	21.91	0.2063
	Visit 4 (day 30 + 2)	23.76	24.06	0.7249
	Visit 5 (day 60 + 2)	22.73	29.2	<0.0001
Health QoL	Baseline visit	17.7	18.14	0.5678
	Visit 4 (day 30 + 2)	19.3	25.14	<0.0001
	Visit 5 (day 60 + 2)	18.3	25.6	<0.0001
Emotional QoL	Baseline visit	21.33	20.63	0.2751
	Visit 4 (day 30 + 2)	17.09	25.83	<0.0001
	Visit 5 (day 60 + 2)	17.97	27.31	<0.0001
Sexual QoL	Baseline visit	10.15	10.29	0.7993
	Visit 4 (day 30 + 2)	9.67	12.4	<0.0001
	Visit 5 (day 60 + 2)	7.97	13.8	<0.0001
Total QoL	Baseline visit	70.09	70.97	0.5161
	Visit 4 (day 30 + 2)	69.82	87.43	<0.0001
	Visit 5 (day 60 + 2)	66.97	95.91	<0.0001

**Figure 3:**
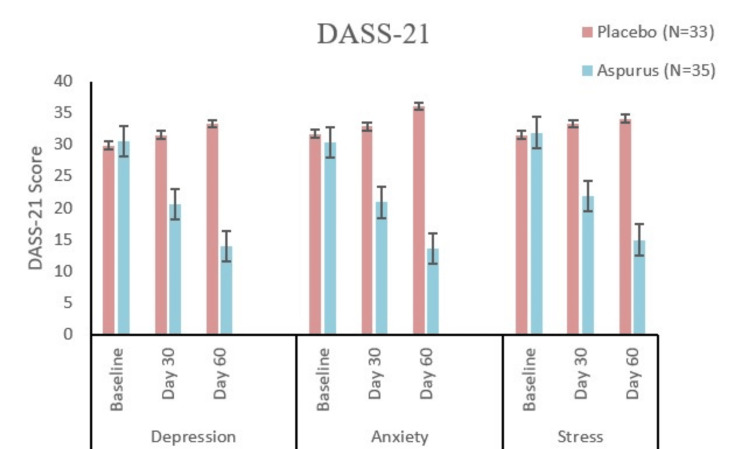
Depression, Anxiety and Stress Scale 21 Items score at baseline, day 30, and day 60 visits for the evaluation of depression, anxiety, and stress for the placebo and active groups. Comparative presentation of the DASS 21 score at various data collection time points for the assessment of depression, anxiety, and stress.

The active group was found to be better in therapeutic responses compared to the placebo (MCC) group (Table 2, Figure 3) in each of the Depression, Anxiety, and Stress scores of DASS-21 with a p-value of 0.5708, 0.0938, and 0.6868, respectively, at the baseline visit to statistically significant outcomes (p < 0.0001) at the final visit (visit 5).

**Table 2:** Summary statistics of Depression, Anxiety and Stress Scale 21 Items questionnaire. Presentation of the depression, anxiety, and stress evaluation outcomes at the baseline, day 30, and day 60 (final visit) visits.

Summary statistics of DASS-21 questionnaire				
Parameter	Visit	Placebo (N = 33)	Active (Aspurūs) (N = 35)	Intergroup P-Value
Depression	Baseline visit	29.88	30.51	0.5708
	Visit 4 (day 30 + 2)	31.52	20.57	<0.0001
	Visit 5 (day 60 + 2)	33.27	14	<0.0001
Anxiety	Baseline visit	31.7	30.34	0.0938
	Visit 4 (day 30 + 2)	32.85	20.91	<0.0001
	Visit 5 (day 60 + 2)	36.06	13.6	<0.0001
Stress	Baseline visit	31.52	31.89	0.6868
	Visit 4 (day 30 + 2)	33.27	21.89	<0.0001
	Visit 5 (day 60 + 2)	34.12	14.97	<0.0001

An intragroup comparison suggested a significant change (p < 0.0001) in the mean Depression, Anxiety, and Stress score of DASS-21 in the active group.

Regensburg Insomnia Scale (RIS): total score evaluation

**Figure 4:**
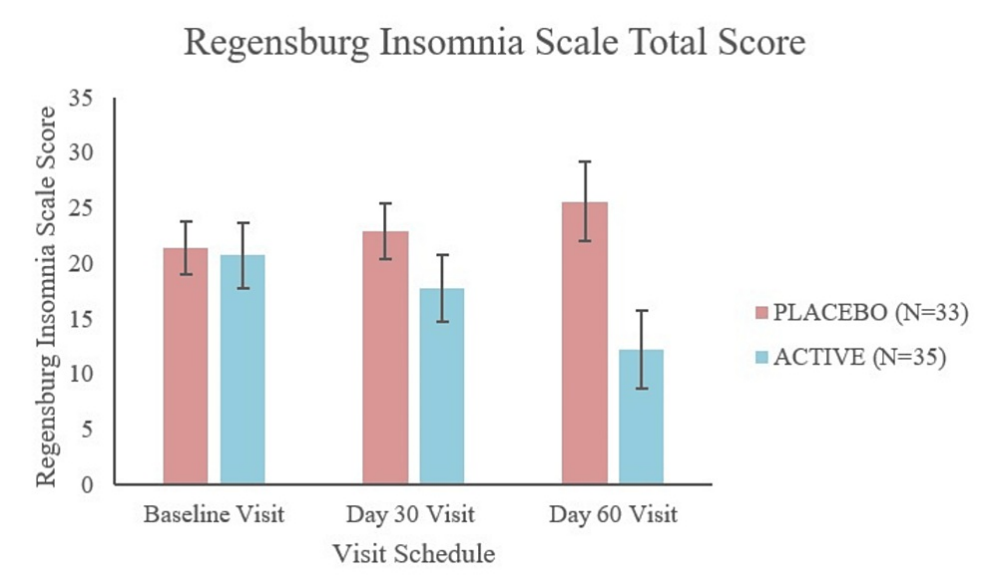
Total RIS score recorded at the baseline, day 30, and day 60 visits. RIS = Regensburg Insomnia Scale

The active group was found to be better in the therapeutic response compared to the placebo (MCC) group in the total RIS score (Table 3, Figure 4). There was a significant change in the total RIS score with a comparative p-value of 0.3222 at the baseline visit to a significant outcome (p < 0.0001) at the final visit (visit 5). The absolute mean change from the baseline score was found to be -8.54 (-41%) in the active group when compared to 4.18 (19.56%) in the placebo (MCC) group.

**Table 3:** Summary statistics of the Regensburg Insomnia Scale (RIS) based on the evaluation of the parameters (poor sleep depth, poor sleep quality, fearful focus on insomnia, hypnotics, and poor daytime functioning) for the placebo and test groups recorded at the baseline, day 30, and day 60 visits.

Summary statistics of the Regensburg Insomnia Scale (RIS)				
Parameter	Visit	Placebo (N = 33)	Active (Aspurūs) (N = 35)	Intergroup P-value
Poor sleep depth	Baseline visit	6.57	6.4	0.5774
	Visit 4 (day 30 + 2)	7.3	5.54	<0.0001
	Visit 5 (day 60 + 2)	8.33	3.94	<0.0001
Poor sleep quality	Baseline visit	6.81	6.85	0.8951
	Visit 4 (day 30 + 2)	7.33	6	<0.0001
	Visit 5 (day 60 + 2)	8.54	3.88	<0.0001
Fearful focus on insomnia	Baseline visit	4.69	4.51	0.6422
	Visit 4 (day 30 + 2)	5.03	3.85	0.0018
	Visit 5 (day 60 + 2)	5.82	2.71	<0.0001
Hypnotics and poor daytime functioning	Baseline visit	3.33	3	0.1521
	Visit 4 (day 30 + 2)	3.3	2.4	<0.0001
	Visit 5 (day 60 + 2)	2.91	1.68	<0.0001
Total score	Baseline visit	21.42	20.77	0.3222
	Visit 4 (day 30 + 2)	23	17.8	<0.0001
	Visit 5 (day 60 + 2)	25.61	12.23	<0.0001

There was a significant change in the poor sleep depth, poor sleep quality, and hypnotics and poor daytime functioning with a comparative p-value of 0.5774, 0.8951, and 0.1521, respectively, at the baseline visit to a significant outcome (p < 0.0001) at both visit 4 and the final visit (visit 5). Moreover, for the fearful focus of insomnia, the change in the p-value was from 0.6522 at the baseline visit to a significant outcome at visit 4 (p = 0.0018) and the final visit (visit 5) (p < 0.0001).

These results show that sleep quality, sleep depth, fearful focus on sleep and hypnotics, and poor daytime functioning improved in the active group compared to the placebo (MCC) group.

The results of RIS also showed significant improvement concerning the time to fall asleep and duration of sleep. In the active group, the study participants took less time to fall asleep. Moreover, they slept for a longer time when assessed at visit 5 compared to the assessment outcomes at the baseline visit. This shows a positive response to the test product evaluated in this study.

Hot flashes and night sweats

A five-point Likert scale was used to measure these secondary outcomes of hot flashes and night sweats. Hot flashes are the most common menopausal symptom which is a sudden feeling of warmth in the body. In the placebo group, out of 33 participants, 19 (57.57%) experienced hot flashes by the final visit (visit 5), whereas, in the active group, out of 35 participants, only four (12.12%) experienced hot flashes by the final visit (visit 5). The absolute mean number of hot flashes in the placebo group reduced from 2 per day at the baseline visit to 1.18 per day at the final visit (visit 5). On the other hand, in the active group, it reduced from 1.97 per day to 0.14 per day. This reduction of hot flashes from 1.83 per day in the active group compared to 0.82 per day in the placebo group clearly shows that the active group is in therapeutic response compared to the placebo group.

There was a significant change (p < 0.0001) in the hot flashes observed in the intergroup comparison at the baseline visit evaluation to the final visit (visit 5). This shows that most participants had a significant reduction in both the frequency and number of hot flashes per day.

Evaluation of the night sweats in the placebo group revealed that 17 participants reported experiencing night sweats at the final visit (visit 5), whereas 27 participants reported having night sweats during the baseline visit. On the contrary, analysis of the active group suggested that 16 participants reported experiencing night sweats during the baseline visit, whereas only two participants reported having night sweats during the last visit (day 60). Further, the absolute mean number of night sweats in the placebo group reduced from 1.52 per day at the baseline visit to 1.06 per day at the final visit (visit 5). On the other hand, in the active group, it reduced from 0.77 per day to 0.06 per day. This reduction of night sweats from 0.71 per day in the active group compared to 0.46 per day in the placebo group shows that the active group had a better therapeutic response compared to the placebo group.

There was a significant positive change in the night flashes with a comparative intergroup p-value of 0.002 at the baseline visit to the final visit (visit 5) outcomes (p < 0.0001). Hence, most participants had a significant reduction in both the frequency and number of night sweats per day. However, the intergroup comparison of “how much did it affect your health status” was not statistically significant, although the frequency was reduced.

The absolute mean number of anxiety/nervousness/feelings of worrying in the placebo group reduced from 2.36 per day at the baseline visit to 1.06 per day at the final visit (visit 5). On the other hand, in the active group, it reduced from 2.57 per day to 0.34 per day. This reduction of anxiety/nervousness/feeling of worrying from 2.23 per day in the active group compared to 1.3 per day in the placebo group suggests that the active group was a better therapeutic responder compared to the placebo group.

There was a significant change in the anxiety/nervousness/feeling of worrying with a comparative intergroup p-value of 1 at the baseline visit to the outcome assessment at the last visit (visit 5) (p < 0.0001). Therefore, the outcomes suggest that most participants had a significant reduction in both the frequency and number of anxiety/nervousness/feeling of worrying episodes per day. Moreover, the intergroup comparison of “how much did it affect your health status” was also statistically significant from the baseline visit results to the final visit (visit 5) outcomes (Table 4).

**Table 4:** Summary of changes in the individual items scores at the baseline, day 30, and day 60 visits.

Summary statistics of the Likert scale				
Parameter	Visit	Placebo (N = 33)	Active (Aspurūs) (N = 35)	Intergroup P-value
Hot flashes	Baseline visit	33	35	1
	Visit 4 (day 30 + 2)	24	5	<0.0001
	Visit 5 (day 60 + 2)	19	4	<0.0001
Night sweats	Baseline visit	27	16	0.0026
	Visit 4 (day 30 + 2)	18	4	<0.0001
	Visit 5 (day 60 + 2)	17	2	<0.0001
Anxiety/Nervousness/Feeling of worry	Baseline visit	33	35	1
	Visit 4 (day 30 + 2)	21	6	0.0027
	Visit 5 (day 60 + 2)	21	6	<0.0001
Fatigue/General weakness with tiredness	Baseline visit	33	35	1
	Visit 4 (day 30 + 2)	23	3	<0.0001
	Visit 5 (day 60 + 2)	23	2	<0.0001
Insomnia/Sleeplessness/Disturbed sleep	Baseline visit	33	35	1
	Visit 4 (day 30 + 2)	27	6	<0.0001
	Visit 5 (day 60 + 2)	27	5	<0.0001

The absolute mean number of fatigues in the placebo group reduced from 2.64 per day at the baseline visit to 1.03 per day at the final visit (visit 5). On the other hand, in the active group, it reduced from 2.69 per day to 0.06 per day. This reduction of fatigue from 2.63 per day in the active group compared to 1.61 per day in the placebo group suggested that the active group had a better therapeutic response compared to the placebo group. Further, a significant change in fatigue with a comparative intergroup p-value of 1 at the baseline visit to the final evaluation outcomes (p < 0.0001) was recorded at the final visit (visit 5). Hence, the outcomes indicate that most participants had a significant reduction in both the frequency and the number of fatigue episodes per day (Table 4).

Similarly, insomnia/sleeplessness/disturbed sleep, mood swings/sudden changes of mood loss of libido, and urinary incontinence significantly reduced in the active group when compared to the placebo group. Therefore, the treatment group had a better outcome for the less affected sleep, improved interpersonal relationships, and improved sexual relationships with their partners.

Vaginal dryness that causes the sensation of burning/itching of the vagina was experienced by fewer study participants in the active group than in the placebo group. The results were similar for bladder weakness and urinary incontinence where fewer participants experienced them in the active group.

One of the secondary outcomes of the study was the assessment of change in the mean of serum estradiol and serum progesterone. The active group had a better therapeutic response compared to the placebo group for serum estradiol and progesterone with a significantly better result at the final visit (serum estradiol: p = 0.0025; progesterone: p = 0.0005) compared to the results during the baseline visit (serum estradiol: p = 0.8983; progesterone: p = 0.3644). In both the active and placebo groups, there was no significant change in the mean value for the serum estradiol and progesterone at the final visit (visit 5) compared to the baseline visit. Thus, the balance of hormones had a positive effect on the regulation of the HPO axis and decreased stress in the participants.

**Figure 5:**
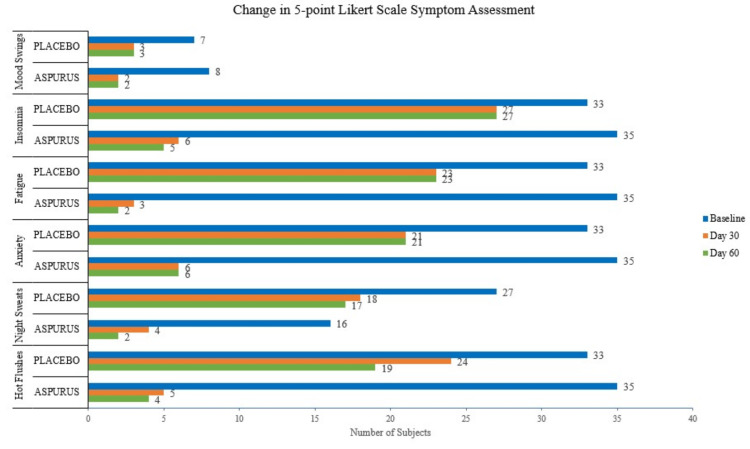
Summary of the five-point Likert scale. A comparative presentation of the outcomes recorded for the parameters (hot flashes, night sweats, anxiety, fatigue, insomnia, and mood swings) was obtained for the placebo and test groups at the baseline, day 30, and day 60 visits.

The treatment satisfaction was better in the active group than the placebo group which can be attributed to the positive effects of the test product evaluated in the present study for menopausal symptoms (Figure 5).

Safety results

Overall, nine participants reported adverse events, with five participants from the active group having experienced six adverse events (dizziness and bloating) and four from the placebo (MCC) group having experienced five adverse events (bloating and nausea). However, all reported adverse events were mild, and all vital parameters were within the physiological range at all visits (Table 5).

**Table 5:** Summary of adverse events in the placebo and active groups. MCC = microcrystalline cellulose

Parameter	Count (N = 68)	
	Placebo group (MCC) (N = 33)	Active group (N = 35)
Have patients experienced adverse events?		
Yes	5 (15.15%)	4 (11.43%)
No	28 (84.84%)	31 (88.57%)
If yes, specify		
Dizziness	0 (0.00%)	1 (2.86%)
Bloating	5 (15.15%)	4 (11.43%)
Nausea	1 (3.03%)	0 (0.00%)

Moreover, it was observed that none of the adverse events were mild. Hence, no action was taken on the study treatment administration because of the adverse events. The vital signs and other observational data were measured as part of the clinical study. Systolic blood pressure, diastolic blood pressure, pulse rate, body temperature, and respiratory rate were measured. There were no significant changes in the above vitals in both groups.

The assessment of treatment satisfaction using the MS-TSQ also yielded statistically significant results within and between treatment group comparisons (Table 6).

**Table 6:** Assessment of treatment satisfaction using the Menopause Symptoms Treatment Satisfaction Questionnaire (MS-TSQ). MCC = microcrystalline cellulose

Parameter	Placebo group (MCC) (N = 33)	Active group (N = 35)
During the past four weeks, how satisfied have you been with the study medication?		
Visit 4 (Day 30 + 2)		
Extremely satisfied	00 (00.00%)	8 (22.86%)
Satisfied	4 (12.12%)	20 (57.14%)
Neutral	19 (57.57%)	7 (20.00%)
Dissatisfied	10 (30.30%)	00 (00.00%)
Extremely dissatisfied	00 (00.00%)	00 (00.00%)
Visit 5 (Day 60 + 2)		
Extremely satisfied	00 (00.00%)	15 (42.86%)
Satisfied	2 (6.06%)	15 (42.86%)
Neutral	22 (66.66%)	5 (14.29%)
Dissatisfied	9 (27.27%)	00 (00.00%)
Extremely dissatisfied	00 (00.00%)	00 (00.00%)

## Discussion

Menopause is a normal physiological condition represented by imbalanced hormonal levels and causes menopausal symptoms such as hot flashes, night sweats, anxiety, and depression in women. In the domains of physical, cognitive, behavioral, and emotional functioning, optimal healthcare requires a definitive way to measure and monitor the associated changes. To understand the effect of the test product on menopausal symptoms, measures such as the total UQoL [14], DASS-21 [15], and the five-point Likert scale were incorporated in this study [16].

The results of this study showed that the subjects in the active group had a significant change in vasomotor symptoms with reduced daily hot flashes, night sweats, anxiety, and depression along with improvement in the physical, mental, and sexual health criteria. The vitals were unchanged when reported pre and post-supplementation of the formulation. The primary outcome measure, i.e., changes in the total UQoL was better in the active group compared to the placebo group.

Middle-aged women were considered as the ideal study participants in this study. We found out that, in many cases, the QoL scores were suboptimal and below the expected scores. Furthermore, the individual QoL scores were below average in sexual domains compared to occupational, emotional, and health QoL scores. The significant predictors of poor QoL in this clinical study were depression and anxiety. This poor QoL can be attributed to the personal circumstances of the women such as unemployment, problems in managing the children, interpersonal relationships, and family issues. Our study observations and the outcomes were similar to a previous study from India which reported poor QoL in 37.2% and 40% peri and postmenopausal women [17].

The assessment of the individual UQoL, i.e., emotional, sexual health, and occupational health, showed significantly better outcomes in the active group than in the placebo group. One of the reasons for having a better QoL in the active group was the effect of the test product on the stressors. The main ingredient in test product formulation has phytoestrogenic properties which can effectively balance hormonal fluctuations and act on stressors, thereby improving the QoL [18].

These phytoestrogens have the unique property of regulating the ovarian cycle in mammals and can aid in either fertility or perimenopausal symptoms and reduce adverse vasomotor symptoms [19,20].

Secondary outcomes such as hot flashes, night sweats, loss of libido, and vaginal dryness were measured using the five-point Likert scale, an ideal measure for larger questionnaires with multiple questions or statements. The accuracy of the scale was high and can be filled quickly.

The outcomes of the five-point Likert scale improved in the active group than in the placebo group, especially for symptoms such as hot flashes, night sweats, anxiety/nervousness/feeling of worrying for the participants, fatigue/general weakness with tiredness, insomnia/sleeplessness/disturbed sleep, mood swings/sudden changes of mood, and loss of libido.

Although hot flashes were present in all participants in both groups, by the end of the final visit, 19 patients in the placebo group and four patients in the active group had hot flashes. This decrease in hot flashes in the active group may be attributed to the hormone-balancing effect of the formulation and the presence of phytoestrogens. Another reason for better response in the active group may be attributed to the adaptogenic nature of the formulation or test ingredient that may help better stress adaptability and stress response. As stress is a major trigger for vasomotor symptoms, stressed women are more likely to have hot flashes.

An earlier study determined that *Asparagus racemosus* is rich in phytoestrogens and effective in reducing menopausal symptoms [19]. Phytoestrogens compete with estrogen to bind with estrogen receptors. Symptoms of menopause are generally caused by the body’s withdrawal from estrogen which is compensated by phytoestrogens which occupy vacant estrogen receptors and stimulate estrogenic action.

Takanari et al. (2016) evaluated the effectiveness of an enzyme-treated *Asparagus racemosus *extract on lowering psychological stress parameters in healthy people and found that it had positive effects on reducing fatigue and dysphoria, enhancing sleep quality, and raising salivary IgA levels in response to stress [21].

We observed a significant loss of libido in the test group. The results are similar to a study by Singh and Kulkarni in 2002 in which a polyherbal formula containing *Asparagus racemosus* was found to be effective in reducing vasomotor symptoms of menopause [22]. According to an animal model study conducted by Jashni et al. in 2016, a dose-dependent aqueous extract of *Asparagus racemosus* significantly stimulated the secretion of gonadotropin-releasing hormone, follicle-stimulating hormone (FSH), luteinizing hormone (LH), estrogen, and progesterone hormones which improved sexual inactivity [23].

The active group showed a significant change in insomnia. The study participants who had disturbed sleep or sleeplessness had significantly improved sleep after the use of this test formulation. Few studies [24-26] have suggested that *Asparagus racemosus* can help treat certain central nervous system disorders and improve sleep. Velavan et al. explored the adaptogenic and nootropic properties of *Asparagus racemosus* in rodents [27]. This neuro-nutraceutical potential can help menopausal women in combating stress-induced anxiety and depression [28,29]. This property can also help in treating insomnia/sleeplessness in women.

Similarly, vaginal dryness was significantly reduced in the active group which can be attributed to the hormone-balancing effect of the test product formulation which would increase vaginal lubrication and keep the vagina dry-free [30].

The laboratory parameters, i.e., serum estradiol and progesterone, were significantly improved in the active group. This result had a positive effect on the regulation of the HPO axis. This balance of hormones aids in the positive feedback of FSH and LH secretion, in turn, regulating ovarian function. Estrogens influence HPA function by modulating adrenal, anterior pituitary, and hypothalamic functions. Stress also causes an imbalance in hormone levels and may cause dysregulation of the HPO axis. In our study, the test product was found to decrease stress and balance hormonal levels, thereby regulating the HPO axis.

Assessment of the MS-TSQ also yielded statistically significant results within and between treatment group comparisons, as observed by others. This can be attributed to the positive effect of the experimental product on the symptoms of menopause. As the symptoms were significantly decreased in most patients without showing any serious adverse events, the satisfaction level of the treatment increased.

Study limitations

A small sample size with specific age groups, participants with varying stages of severity in symptoms, and fluctuating estradiol levels of the study participants may mask some of the study outcomes. Hence, a larger sample size, multicenter study with diverse samples, and inclusion of diverse age groups may help generalize and confirm the present observations.

## Conclusions

Most studies conducted so far are on postmenopausal women, whereas our study was conducted on both pre and postmenopausal women. Moreover, very few studies have shown the safety and efficacy of Shatavari alone on humans. The findings from this study support the positive effects of a novel Shatavari formulation taken for 60 days at a dose of 250 mg twice a day. This study also showed that the test product was safe and well-tolerated with no major adverse events in the participants with menopausal symptoms (hot flashes, anxiety, stress, sleep, etc.). Therefore, it could be a safe alternative to modern drugs. The efficacy of the test product was well demonstrated concerning a significant improvement in menopausal symptoms. It was found to be an effective therapy for psychological and somatic problems related to menopausal syndrome. The findings of our study supported the traditional use of Shatavari with modern scientific evidence. Further, clinical and pharmacological studies with longer durations and larger sample sizes are required to understand the mechanistic actions of this formulation in menopausal women.
